# Utilizing Upcycled Graphene Nanoplatelet-Coated Glass Fibers as a Performance Booster and Compatibilizer for Enhanced Mechanical Performance of Polypropylene/Glass Fiber/Graphene Nanoplatelet Composites

**DOI:** 10.1021/acsomega.4c00214

**Published:** 2024-04-02

**Authors:** Gülayşe Şahin Dündar, Burcu Saner Okan

**Affiliations:** † Department of Materials Science and Nano Engineering, Faculty of Engineering and Natural Sciences, Sabanci University, Orhanli-Tuzla, Istanbul 34956, Turkey; ‡ Sabanci University Integrated Manufacturing Technologies Research and Application Center & Composite Technologies Center of Excellence, Teknopark Istanbul, Pendik, Istanbul 34906, Turkey

## Abstract

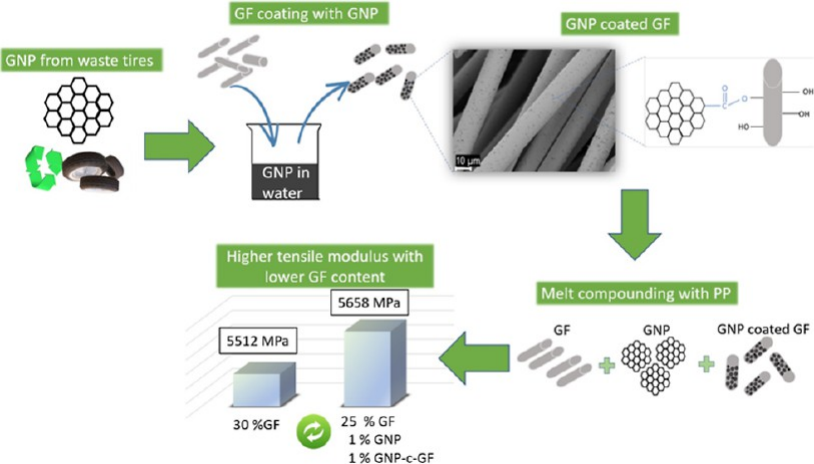

The high usage ratio and elevated density of glass fibers (GFs), often surpassing twice that of polymers, can contribute to undesired increases in the overall density of polymeric materials. One potential solution is the incorporation of graphene as a secondary additive, offering a lower specific gravity and exceptional mechanical properties. In light of this, waste tire-derived graphene nanoplates (GNPs) were optimized for coating onto GFs by considering factors such as surface treatment of the fiber, the dispersion quality of GNP, and the coating technique. The resulting GNP-coated GF (GNP-c-GF) was initially incorporated into pure polypropylene (PP) at low weight percentages (0.1–1 wt %), and 31% increase in the tensile modulus was achieved compared to neat PP. Subsequently, 1 wt % GNP-c-GF was utilized as a compatibilizer in PP/GF/GNP composites to enhance the compatibility between GNP and GF. By strategically incorporating GNP and GNP-c-GF at a lower GF ratio, the detrimental impact on the tensile modulus of 30% GF-filled PP was effectively mitigated, leading to a remarkable enhancement to an impressive value of 5658 MPa. The successful integration of GNP and GNP-c-GF exemplifies their promising potential as additives for achieving superior mechanical properties in composite materials, while concurrently promoting the utilization of recycled content.

## 1 Introduction

Extensive research has been conducted by numerous scholars exploring the utilization of polymer composites incorporating both natural and synthetic fibers.^1^ Within polymer/fiber composites, glass fiber (GF) is the most preferred reinforcement material despite its limitations.^2,3^ GF-reinforced polypropylene (PP) composites are extensively employed across various applications owing to their superior mechanical and thermal characteristics, as well as their relatively cost-effective nature in comparison to alternative fiber types such as carbon and aramid.^4^ But in most cases, high loadings of GFs ranging from 30 to 50 wt % are required to achieve the desired performances from the material, which leads to yield unfavorable effects such as undesirable increase in specific gravity, brittleness, reduced processability, and diminished melt flow characteristics.^5,6^

Hybrid composites have gained significant popularity in diverse engineering applications due to their ability to showcase novel physical properties and functionalities.^7^ There has been a tendency to produce graphene-based hybrid composites^8^ to tackle encountered problems in the material or when multifunctionality is expected such as higher mechanical and thermal properties, cost reduction, and lightness simultaneously. The remarkable mechanical, electronic, thermal, and magnetic properties of graphene have made it a highly sought-after subject of research in recent times.^9^ In recent investigations, considerable attention has been given to the development of GF/graphene/PP composites, with the objective of augmenting the mechanical, thermal, and electrical attributes of these materials through the integration of graphene within GF-reinforced PP matrices. Papageorgiou et al. investigated the thermal stability and flame retardancy characteristics of PP nanocomposites incorporating graphene nanoplatelets (GNPs), GFs, or a hybrid combination of both fillers, revealing a 57 °C increase in the thermal decomposition temperature of the hybrid composite (PP-GF16-GNP20) compared to neat PP and individual filler compositions.^100^ In another study, Ghorbani et al. prepared hybrid nanocomposites with PP, GF, and exfoliated GNP, showing improved fiber–matrix adhesion. The inclusion of 1 and 2 wt % graphene in samples containing 10 wt % GF increased Young’s modulus by 16 and 21%, respectively.^10^ While GF/graphene/PP composites offer various advantages, there are also some drawbacks and challenges associated with their development and application. Ensuring strong interfacial adhesion between the GF, graphene, and PP matrix is crucial for optimal composite performance. However, the presence of certain constraints such as the absence of reactive functional groups on the fiber surface and poor wettability adds complexity to the task.^11^ Additional steps, such as functionalization, mixing, and processing, are required to ensure proper dispersion and interfacial bonding. To achieve better dispersion and interfacial bonding between graphene, GF, and the PP matrix, researchers have explored various surface modification techniques.^12^ Surface functionalization of graphene and GF can enhance their compatibility with the PP matrix, leading to improved interfacial adhesion and overall composite performance. While the research on GF/graphene/PP composites has shown promising results, the transition from laboratory-scale experiments to industrial-scale production can present challenges since graphene is still relatively expensive compared to other reinforcing materials, and therefore, the high cost of graphene can limit its widespread use in industrial applications, especially in large-scale production. Scaling up the production process while maintaining the desired properties and cost-effectiveness requires further optimization and process engineering. The introduction of graphene derivatives onto the fiber surface in recent years has proven to be an effective method for improving the interfacial properties of composites, resulting in increased specific surface area and stronger chemical bonding between the fiber and the matrix.^13^ Researchers and engineers in the field of composite materials have been exploring various coating techniques and formulations to successfully incorporate graphene onto GF for improved performance in a range of applications.^14^ While coating GNP onto GF may require careful consideration and optimization, it is an achievable process with the potential to enhance the properties of the resulting composite. Generally graphene oxide have been used to coat GFs by synthesizing from graphite with the Hummers’ method which includes several steps with a variety of chemicals.^14,15^ These extra processing steps can increase the production cost and complicate the scaling-up of manufacturing processes. However, in today’s culture, there is a tremendous need for materials that are lightweight, strong, and inexpensive.^16^ These challenges are being actively addressed by researchers and industry professionals, and ongoing studies aim to overcome these drawbacks and improve the performance and practicality of GF/graphene/PP composites.

Herein, a facile and practical coating technique was employed to treat GFs with upcycled GNP derived from waste tires. The utilization of waste tire-derived GNPs aligns with sustainable practices, addressing both environmental concerns and resource efficiency. This strategic approach not only contributes to the development of advanced materials but also promotes a more environmentally conscious and economically viable solution for industrial applications. The GNPs were utilized without any modifications, and a dip coating optimization study was conducted by dispersing them in water at various concentrations. The resulting GNP-coated GFs (GNP-c-GF) were subsequently incorporated into pure PP and PP/GF/GNP composites to comprehensively evaluate their mechanical, morphological, and flow properties. The interfacial compatibility between GNPs, GFs, and the PP was greatly improved by incorporating a small quantity of 1% GNP and 1% GNP-c-GF into the composites with varying GF contents (15, 20, 25, and 30%). Notably, the addition of only 1% GNP-c-GF in PP exhibited a remarkable 31% increase in the tensile modulus compared to neat PP. Moreover, the incorporation of GNP-c-GF into composites containing both GFs and GNPs played a crucial role as a compatibilizer, effectively enhancing the homogeneity of the composite system and promoting a uniform dispersion within the matrix. This strategy facilitated the attainment of enhanced performance by utilizing a compatibilizer comprising the inherent constituents of the composite, thereby circumventing the need for the incorporation of a separate structural element.

## 2 Experimental Section

### 2.1 Materials

E-type GFs coated with a silane-based sizing with a strand length of 4 mm supplied from Johns Manville. Homo PP with a density of 905 kg/m^3^ intended for injection molding was supplied from BOREALIS. Sulfuric acid with a purity of 99% and (30 wt %) hydrogen peroxide were purchased from Sigma-Aldrich. GNPs were supplied from Nanografen Co., Kocaeli, Turkey. The detailed characterization regarding to upcycled GNP can be found in our previous study.^17^

### 2.2 GF Surface Treatment

In order to remove organic coating from the surface and desize the GF, piranha solution has been used in literature.^18^ Here, a safer piranha solution was used to desize and hydroxylate GF surface with the ratio for H_2_SO_4_/H_2_O_2_ was (6:1) by volume. 10 g of GF was taken into an empty beaker and certain amount of H_2_SO_4_ was added to it. Afterward, H_2_O_2_ was added very slowly, and the reaction took place for 30 min. After cooling solution was filtered, it was washed and dried and coated as hydroxylated GF (h-GF).

### 2.3 Coating GNP onto GF

GNP/water dispersions were prepared in four different concentrations, and 1 h probe sonication was performed. Afterward, 10 g of previously synthesized h-GF was dipped into these dispersions and kept for 1 h for coating. GNP-c-GFs were washed and dried with a mixture of water/ethanol and were coded as GNP-c-GF.

### 2.4 Compounding and Injection Molding of Polymer Composites

All composites were produced by a using COPERION ZSK26 MC twin screw extruder. GNP and GNP-c-GF were premixed with PP granules and fed into the main feeder. GF were fed with side feeding. Test samples were produced by injection molding according to ISO 527 and ISO 178 standards. The injection molding process was conducted by utilizing an Arburg injection molding machine, specifically the Allrounder 320C model, characterized by a clamping force of 500 kN. The homo-PP material underwent melting at a temperature of 210 °C. Notably, a heated mold was employed throughout the molding process, maintaining a temperature of 35 °C.

### 2.5 Characterization and Testing

The functional groups of the GF and GNP-coated samples were analyzed using Thermo Scientific Fourier transform infrared (FTIR) spectroscopy, while X-ray photoelectron spectroscopy (XPS) was employed to monitor their functional groups and chemical compositions. Structural characterization of the coating materials was performed using a Bruker D2 PHASER Desktop diffractometer with Cu Kα radiation (λ = 1.5406 nm) for X-ray diffraction (XRD). To examine the surface morphology, the composite samples were fractured under liquid nitrogen, coated with a thin layer of gold, and observed under a Leo Supra 35VP field emission scanning electron microscope. Mechanical tests were conducted using an Instron 5982 Static Universal Test Machine following the ISO 527-2 and ISO 178 standards for tensile and three-point bending tests.

## 3 Results and Discussion

### 3.1 Structural and Morphological Analysis of GF Hydroxylation and GNP Coating

**Figure 1:**
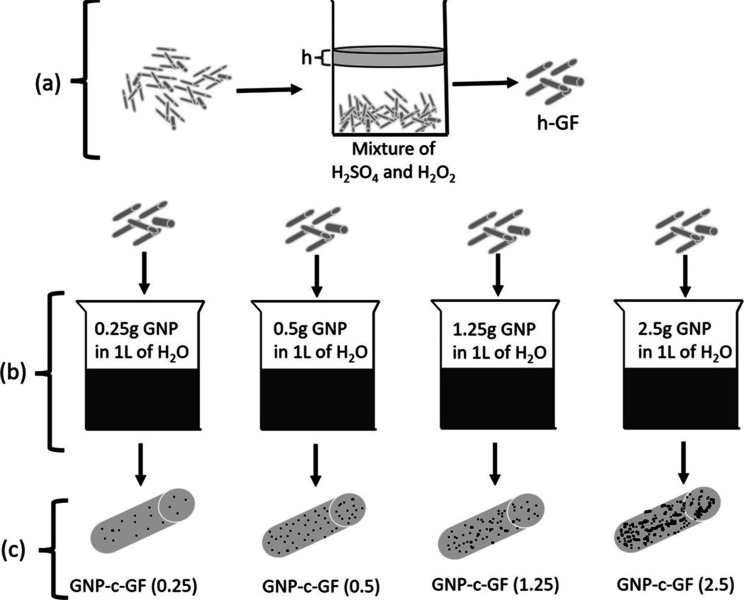
Experimental scheme of (a) desizing and hydroxylation of GF, (b) dip coating of GF with GNP at varying concentrations, and (c) coated samples.

The process of coating GNPs onto GF involves several steps and considerations. While it is feasible to coat GNPs onto GF, the ease of the coating process can depend on various factors. The surface of the GF should be appropriately prepared to ensure good adhesion and bonding between the fiber and GNP coating. Surface treatments, such as cleaning, activation, or functionalization, may be necessary to enhance the bonding between the GNP and the fiber surface. In this context, 6:1 ratio of H_2_SO_4_ and H_2_O_2_ was used for cleaning and hydroxylation of the GF surface. H_2_O_2_ was added dropwise very slowly on the GFs immersed in H_2_SO_4_. The experimental stages of the process are depicted in Figure 1 and the actual experimental picture is provided in Figure S1 (Supporting Information document). As the reaction continued, a layer began to form in the beaker as shown in the Figure 1a as “h”, and the thickness of this layer increased over time. This layer corresponds to transformation of the oxidized and hydroxylated organic surface. After this step, the GFs have the appropriate surface chemistry to be coated with the GNP.

There are various techniques available for coating GNPs onto GF, including dip coating,^19^ spray coating,^20^ electrophoretic deposition,^21^ and resin infusion methods.^22^ The choice of the coating technique depends on factors such as the desired thickness, uniformity, and scalability of the coating. Each technique has its own set of advantages, challenges, and process parameters that must be carefully considered. Dip coating offers several advantages as a technique for coating GNPs onto GF. It is a relatively simple and cost-effective coating technique. It involves immersing the GF into a GNP dispersion or solution and withdrawing it at a controlled speed. It offers control over coating thickness, ensuring consistency and enabling conformal coating. The simplicity and scalability of the dip coating make it suitable for both laboratory- and industrial-scale applications. It is a versatile technique compatible with various types of GNPs and matrix materials. Dip coating also accommodates complex geometries, allowing for uniform coating on intricate surfaces. Dip coating can be applied to GFs of various shapes and complex geometries. Whether it is a continuous fiber, fabric, or 3D structure, dip coating can uniformly coat the entire surface, including crevices and intricate features. This compatibility with complex geometries makes dip coatings suitable for a wide range of GF-based composite applications. In this study, dip coating was applied to coat GNP on GF due to the advantages mentioned. GNP was dispersed in water as shown in Figure 1c, and then h-GFs were immersed in this dispersion for half an hour. In the meantime, the color of the GF surfaces turned black and was washed with a mixture of water and alcohol after the process. The color retention of the GFs after several washes indicates the good stability of the coating. Achieving a well-controlled and consistent GNP coating on GF requires optimization of process parameters such as GNP concentration, coating time, and drying conditions. The optimization process may involve iterative experimentation and characterization to determine the ideal conditions for achieving the desired GNP coating thickness and uniformity. GNPs have a tendency to agglomerate, which can hinder their effective coating onto the GF surface. Therefore, achieving a stable and homogeneous GNP dispersion is crucial for a successful coating. For this, the effectiveness of the coating was measured by preparing separate GNP dispersions at four different concentrations. GNP/water dispersions were prepared by adding 2.5, 1.25, 0.5, and 0.25 g of GNP in 1000 mL of water, respectively. The amount of dispersed GNP was added at the end of the sample codes. For example, GNP-c-GF (2.5) refers to hydroxylated GF coated by dispersing 2.5 g of GNP in 1000 mL of water.

**Figure 2:**
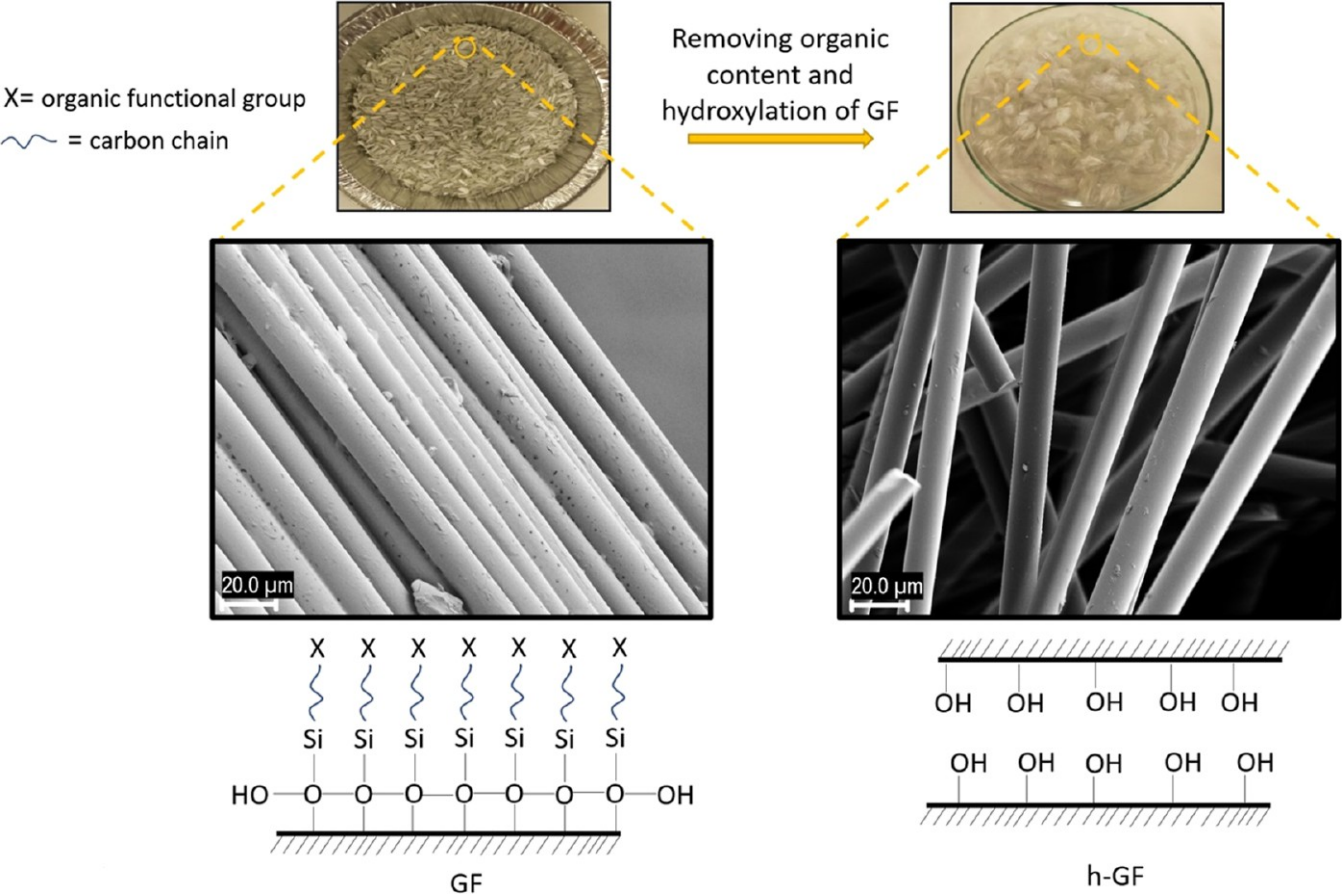
Related SEM images and chemical compositions belong to GF and h-GF.

SEM images in Figure 2 depict the contrasting characteristics of untreated and treated GFs. In the untreated sample, the fibers exhibit interconnectivity with discernible surface coating. However, following the hydroxylation process, the fibers become disconnected, leading to a smoother surface texture and increased separation between the individual fibers. Consequently, the modified GF surface becomes amenable to the deposition of GNP.

**Figure 3:**
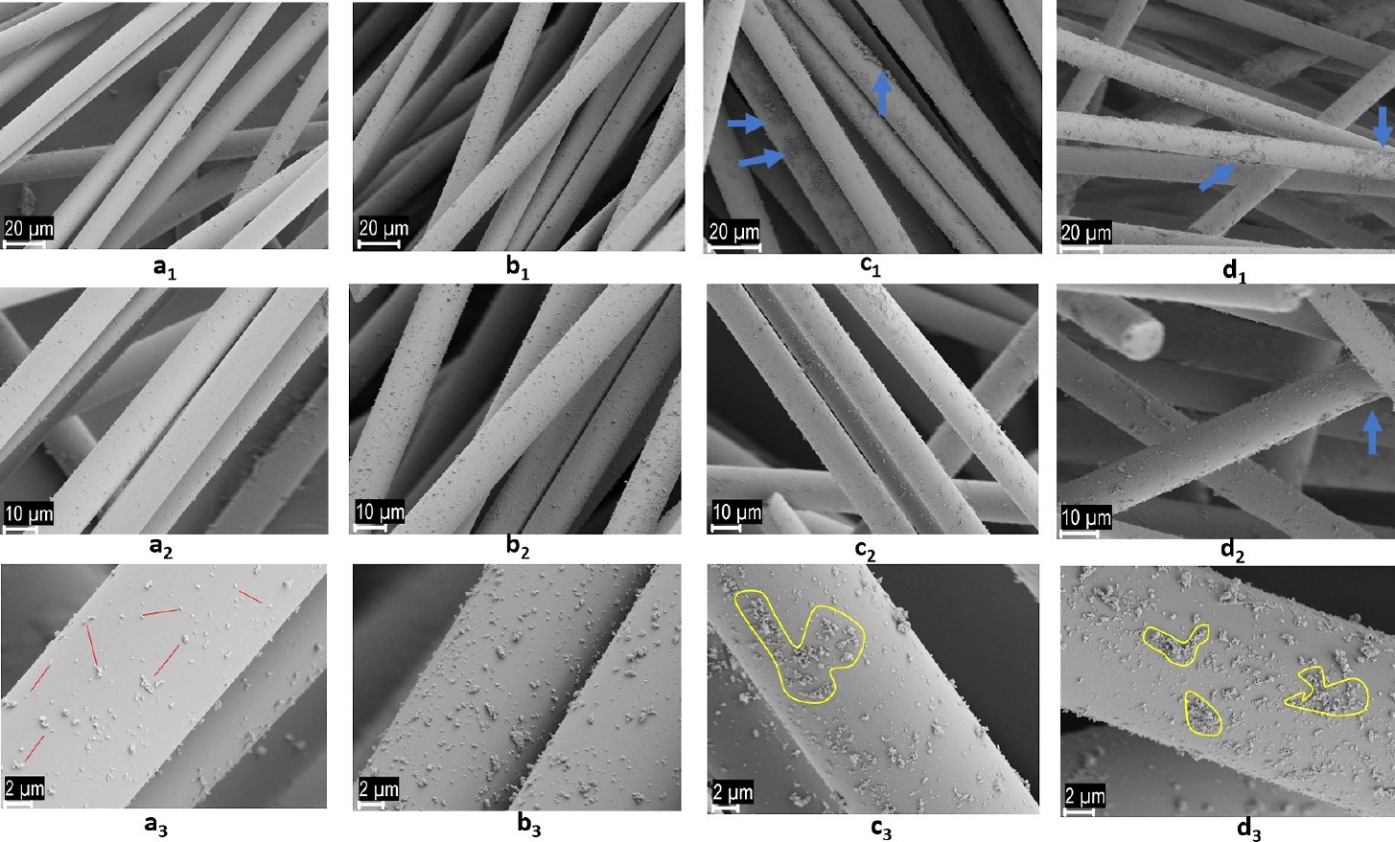
SEM images of (a_1_–a_3_) GNP-c-GF (0.25), (b_1_–b_3_) GNP-c-GF (0.5), (c_1_–c_3_) GNP-c-GF (1.25), and (d_1_–d_3_) GNP-c-GF (2.5) at different magnifications.

The SEM images depicted in Figure 3 exhibit GFs that have been coated with various concentrations of GNP, captured at different magnifications. Initially, it can be inferred that the adhesion of GNPs to the fibers is robust across all concentrations. However, the extent of adhesion and the propensity for agglomerate formation exhibit notable variations. When the punctuated GNPs are classified based on the concentration of the immersion solution, the images corresponding to GNP-c-GF (0.25) are designated as a_1_–a_3_, GNP-c-GF (0.5) as b_1_–b_3_, GNP-c-GF (1.25) as c_1_–c_3_, and GNP-c-GF (2.5) as d_1_–d_3_. Upon close examination of the sample with the highest GNP-c-GF concentration (2.5) (d_1_–d_3_), substantial agglomeration is observed. The GNP layers densely conglomerate in regions marked by blue arrows, resulting in an uneven distribution on the fibers. Further scrutiny of areas demarcated by yellow lines reveals denser clusters, indicating a higher concentration of the immersion solution. Although a reduction in agglomeration is apparent when the concentration is halved, clusters persist, and the distribution of GNPs on the fiber remains nonhomogeneous. Upon halving the concentration of the immersion solution even further (b_1_–b_3_), the prominence of increased agglomerates diminishes, giving rise to a more homogeneous distribution. Remarkably, the formed agglomerates exhibit closer proximity to one another with a relatively consistent interparticle distance. Notably, in columns c and d, agglomerations exceeding 1 μm are absent due to the merging of clusters. Upon further examination of the concentration that has been halved, an increase in the particle distance becomes evident. The gap between each GNP particle is approximately 2 mm, raising doubts regarding the adequacy of GNP adhesion to the fiber. In this case, GNPs were not coated on the GFs but appeared to be interspersed throughout the structure.

**Figure 4:**
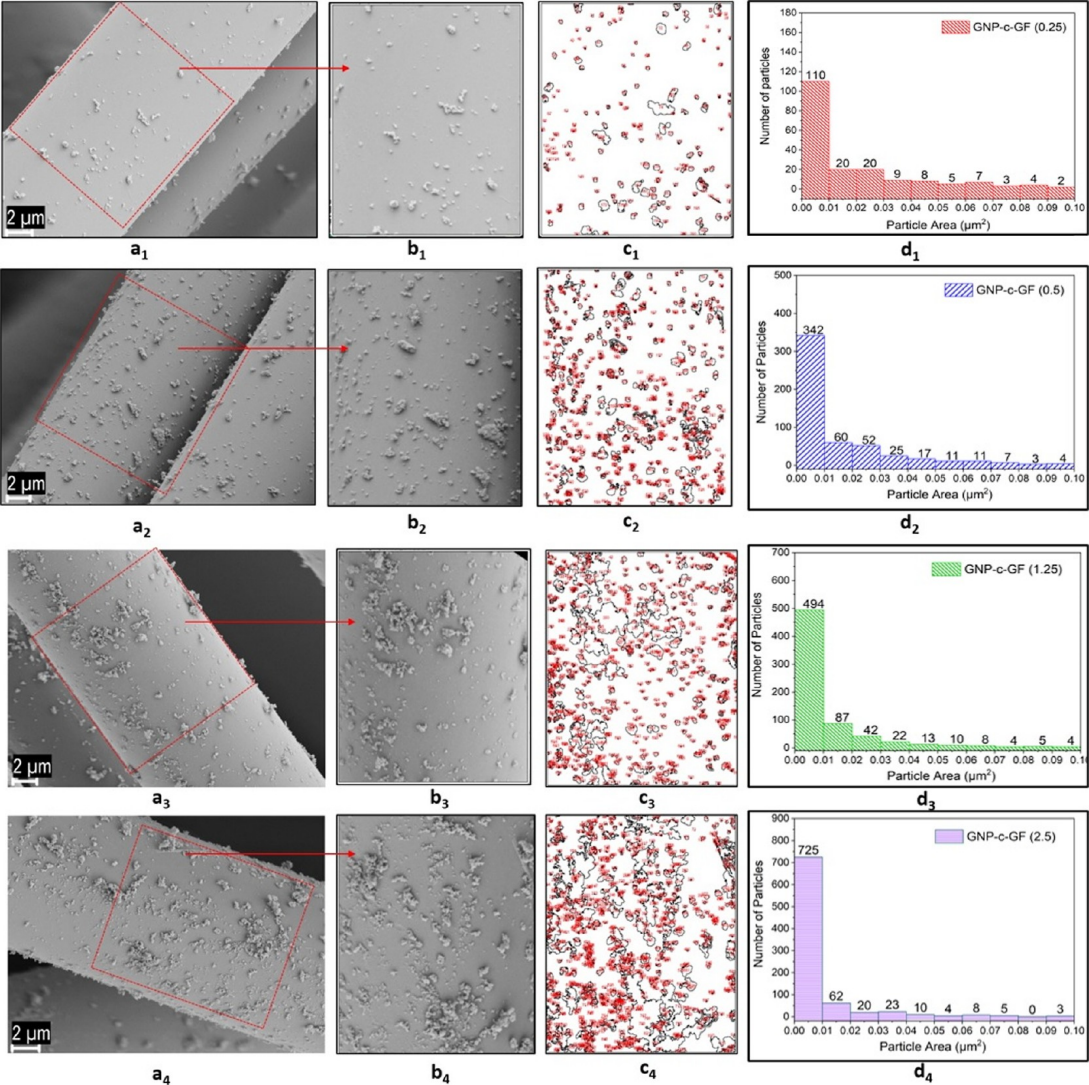
(a_1_–a_4_,b_1_–b_4_) SEM images and (c_1_–c_4_,d_1_–d_4_) particle distribution of GNP-c-GF (0.25), GNP-c-GF (0.5), GNP-c-GF (1.25), and GNP-c-GF (2.5).

Figure 4 and Table 1 illustrate the particle area distribution obtained by calculating particle sizes by using ImageJ software over a uniform area of the coatings. The accompanying table presents the total number of particles and their distribution within specific size ranges. Increasing the concentration of GNPs results in a rise in the proportion of particles below 0.01 μm from 55 to 81%. However, to attain a uniform distribution of particles, it is crucial to take into account a wide range of particle sizes. Notably, GNP-c-GF (0.25) and GNP-c-GF (0.5) exhibit the highest proportions of particles, accounting for 28 and 27%, respectively, within the 0.01–0.05 μm range. It is worth noting that GNP-c-GF (0.25) shows no agglomerates exceeding 0.5 μm, but as mentioned earlier, the interparticle distance is relatively large. On the other hand, GNP-c-GF (0.5) demonstrates a more homogeneous particle distribution as it contains fewer particles exceeding 0.5 μm and a higher number of particles within the 0.01–0.05 μm range compared to other samples. Considering its similar characteristics to GNP-c-GF (0.5) and to avoid excessive GNP usage, GNP-c-GF (1.25) is not selected for incorporation into the composites. Notably, GNP-c-GF (2.5) exhibits visible agglomeration intensity with a significant clustering of particles above 0.5 μm.

**Table 1:** Number of Particles in GNP-c-GF Samples and Their Distribution (%)

sample name	total number of particles	under 0.01 μm	between 0.01 and 0.05 μm	between 0.05 and 0.1 μm	between 0.1 and 0.5 μm	above 0.5 μm
GNP-c-GF (0.25)	202	110 (55)	57 (28)	21 (11)	14 (7)	
GNP-c-GF (0.5)	563	342 (61)	154 (27)	36 (6)	29 (5)	2 (0.4)
GNP-c-GF (1.25)	725	494 (68)	164 (23)	31 (4)	30 (4)	6 (0.8)
GNP-c-GF (2.5)	897	725 (81)	114 (13)	20 (2)	31 (3)	7 (0.8)

**Figure 5:**
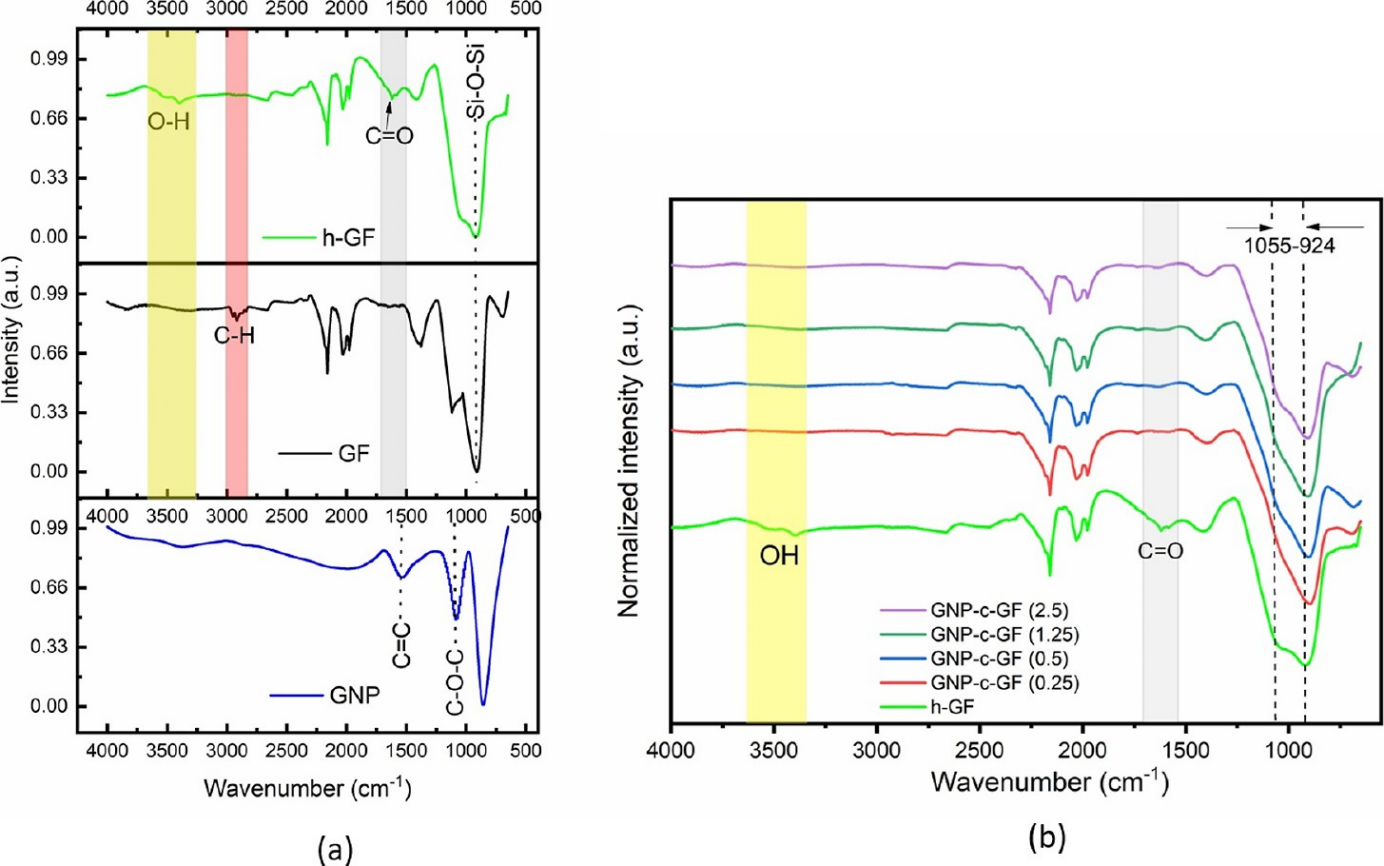
FTIR spectra of (a) GF, h-Gf, and GNP and (b) GNP-c-GF samples.

FTIR spectroscopy enables the identification and analysis of a sample’s composition by studying its infrared absorption patterns. Figure 5a displays the FTIR spectra of GNP, GF, and h-GF. GNP reveals two distinctive peaks at 1550 and 1080 cm^–1^ corresponding to C=C aromatic stretch and C–O–C epoxy groups, respectively^23^ In contrast, the black line in the GF spectrum reveals stretching vibrations of C–H groups in the range of 2918 to 2953 cm^–1^,^24^ indicative of sizing and an organic coating on the purchased GF. Following the hydroxylation process, denoted by the green line, the disappearance of CH peaks in the 2918–2953 cm^–1^ range suggests successful organic group removal from the h-GF surface. Furthermore, a peak at 3400 cm^–1^ corresponds to hydroxyl groups,^25^ while a new peak at 1619 cm^–1^ signifies the formation of C=O carboxylic groups on the fiber surface. This corroborates the effective removal of organic groups from the GF surface through hydroxylation. Peaks at 877–948 cm^–1^ are indicative of Si–O–Si bonds, commonly found in silicon-based materials.^26^ Transitioning to Figure 5b, which illustrates GNP-c-GF samples at varying GNP immersion concentrations, a noteworthy disappearance of peaks at 3400 and 1619 cm^–1^ compared to that of h-GF is observed. This disappearance implies the successful coating of GNP onto GF, facilitated through the epoxy groups of GNP and the hydroxy and carboxyl groups on GF. However, due to overlapping peaks across all samples, specific bond recognition proves challenging. A notable change in absorption behavior is witnessed in the 924–1055 cm^–1^ range of the GNP-c-GF samples, with the disappearance of the 1055 cm^–1^ peak seen in GF and the heightened prominence of the 935 cm^–1^ peak. This phenomenon suggests the incorporation of GNP onto GF, as a similar and robust absorption is observed in GNP within that range. While no distinct differences are discerned based on GNP concentration, the disappearance and emergence of specific peaks substantiate the effective integration of GNP onto GF. Notwithstanding these comprehensive insights, a more detailed elucidation of the chemical bonds and surface functionalities can be attained through additional XPS analysis. XPS is poised to offer a more intricate and comprehensive understanding of the chemical composition and bonding configurations, complementing the wealth of information gleaned from the FTIR chemical bonds.

**Figure 6:**
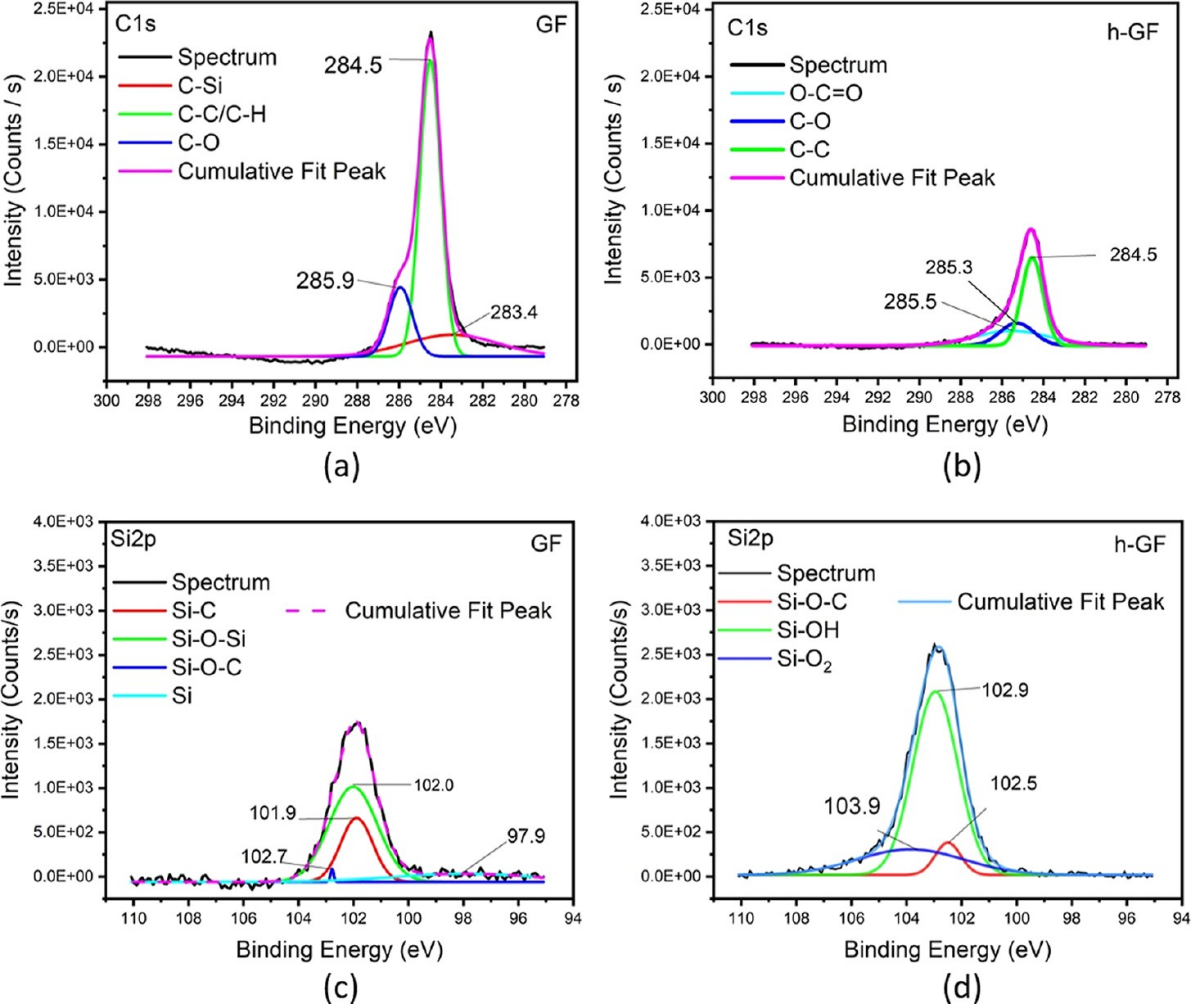
Deconvoluted C 1s of (a) GF and (b) h-GF, and deconvoluted Si 2p of (c) GF and (d) h-GF.

**Figure 7:**
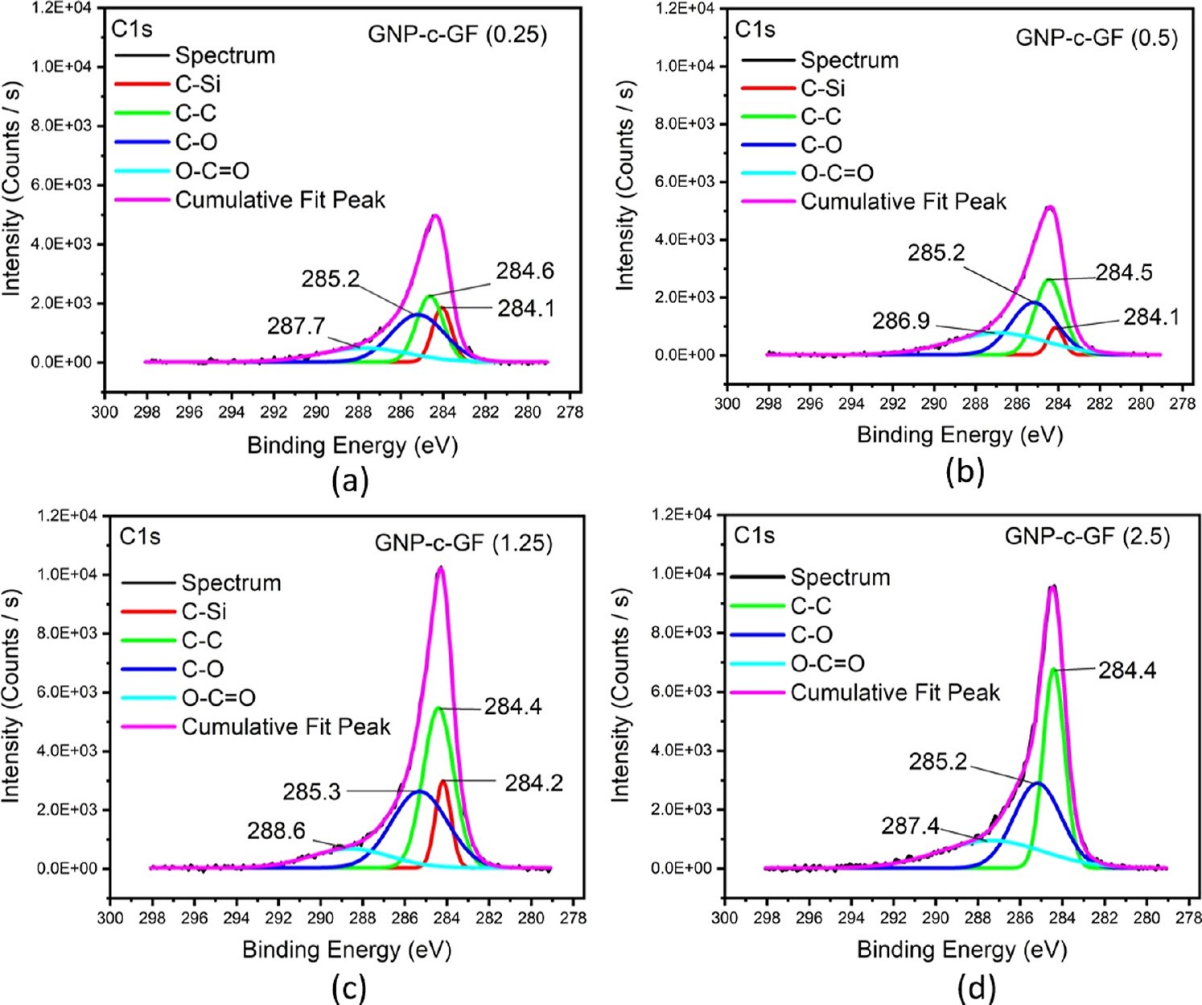
Deconvoluted C 1s of (a) GNP-c-GF (0.25), (b) GNP-c-GF (0.5), (c) GNP-c-GF (1.25), and (d) GNP-c-GF (2.5), respectively.

XPS is a powerful analytical technique used to investigate the chemical composition, bonding states, and electronic structure of materials by bombarding the sample surface with X-rays and detecting the reflected electrons.^27^ XPS plays a crucial role in understanding chemical bonds since it provides information about the elemental composition of a material’s surface.^28^ Table of X-ray survey spectra and related deconvoluted peaks is presented in Table 2, and Figures 6 and 7. According to Table 2, the acquired GF exhibits a carbon content of 69.68%, primarily attributed to the sizing agent commonly employed for matrix compatibility on GFs. XPS survey data demonstrate an approximately 20% reduction in atomic C content (decreasing from 69.68 to 49.08%) after posthydroxylation of GF due to the elimination of organic groups stemming from the sizing process. Following hydroxylation, the Si concentration increases to 17.84%, indicating improved signal collection from the GF surface as a result of the partial removal of the sizing material. As XPS is a surface-sensitive technique, probing the outermost layers of a material (typically a few nanometers), an increased Si concentration on the surface can be reasonably expected, particularly after the removal of organic groups, making Si more detectable on the surface. As XPS measurements probe a specific depth, coating-related peaks become prominent. In-depth examination of GNP-c-GF samples illustrates an elevated carbon content ranging from 32.73 to 43.83% with increasing immersion concentration, suggesting the formation of a thicker and more abundant GNP layer. Additionally, the oxygen content diminishes from 42.7 to 35.45%, while the Si content slightly decreases due to the denser GNP layer formation. Figure 6a,b presents the deconvoluted peaks of C 1s for GF and h-GF, respectively. The peak detected at 284.5 eV corresponds to the C–C and C–H bonds,^7^ exhibiting reduced intensity in Figure 6b, representing h-GF. Conversely, the absence of a peak at 283.4 eV on h-GF, attributed to the C–Si bond from the sizing agent, indicates successful removal of the sizing agent. Likewise, Figure 6c,d depicts the deconvoluted peaks of Si 2p for GF and h-GF, respectively. In Figure 6c, the presence of Si–O–Si bonds, the fundamental constituents of GFs, is observed at 102.0 eV and Si–O–C bond observed at 102.7 eV.^29^ Furthermore, Figure 6d displays the emergence of Si–OH groups at 102.9 eV after hydroxylation. In Figure 7, C 1s spectra of GNP-c-GF samples were presented. C–C peaks in green reveal an incremental intensity from GNP-c-GF (0.25) to GNP-c-GF (2.5), indicating enhanced GNP attachment onto h-GF. The same pattern for the C–O bond (blue) is also observable. The red line that represents the C–Si bond disappeared for GNP-c-GF (2.5) due to the high agglomeration and clustering of GNP particles on the surface.

**Table 2:** XPS Survey Scan Results for GF, h-GF, and GNP-c-GF Samples

sample name	C (atomic %)	O (atomic %)	C/O ratio	Si (atomic %)	others (atomic %)	included other elements
GF	69.68	19.62	3.55	6.57	4.13	Al, N, F
h-GF	49.08	33.07	1.48	17.84	0.01	Al, Ca, N
GNP-c-GF (0.25)	32.73	42.70	0.77	22.39	2.18	Al, S, N
GNP-c-GF (0.5)	36.73	38.16	0.96	20.65	4.46	Al, Ca, N
GNP-c-GF (1.25)	40.92	38.19	1.07	18.66	2.23	Al
GNP-c-GF (2.5)	43.83	35.45	1.24	18.39	2.33	Al, N

**Figure 8:**
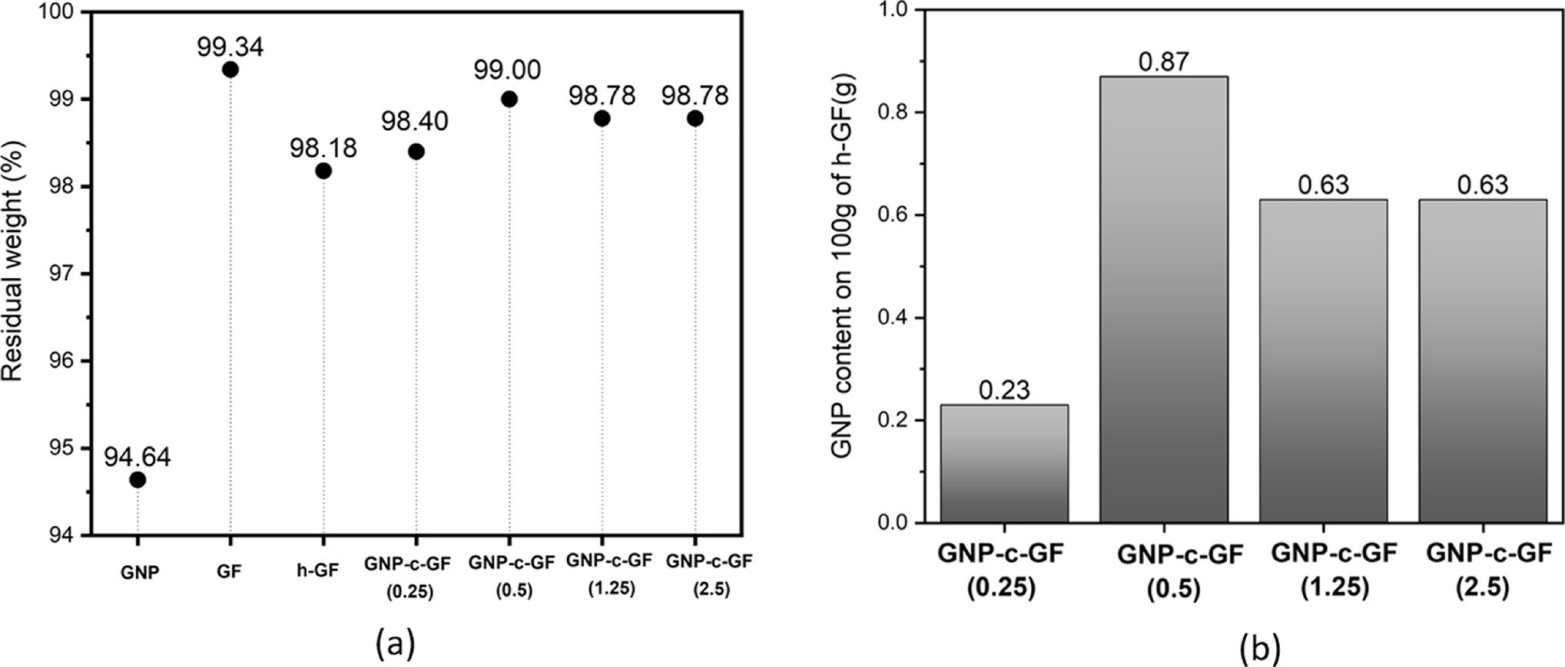
(a) Residual weight of GNP, GF, h-GF, and GNP-c-GF materials. (b) GNP content on GNP-c-GF samples according to TGA.

Thermogravimetric analysis (TGA) is a valuable technique employed to quantify the coating content in diverse materials.^30^ In the context of coatings, TGA allows for the determination of the percentage of coating within a composite material, as it provides quantitative data on mass loss, allowing for the precise measurement of even minor changes in the coating composition. Figure 8a depicts the residual weight of GNP, GF, h-GF, and GNP-c-GF samples, and Figure 8b shows the GNP content on each coating sample. After coating on h-GF in GNP-c-GF (0.25), its residual weight increased from 98.18 to 98.40. In this case, it can be said that the difference of 0.22 is due to the GNP coating. However, since 94.64% of pristine GNP remains intact up to the same temperature, this value of 0.22 is 94.64% of GNP by weight. The GNP content obtained as a result of the calculation made in this approach is shown in Figure 8b for each coating. As can be seen, the least GNP content was found in the GNP-c-GF (0.25) medium, while the highest GNP content was obtained in the GNP-c-GF (0.5) medium. With the further increase of the dipping concentration after this point, the adhesion rate of GNP on h-GF did not increase and much higher agglomeration was obtained with less GNP content. This proves that the GNP concentration of the dipped dispersion and the ratio of GNP attached to the h-GF are not directly proportional and there should be an optimum concentration of GNP/water.

**Figure 9:**
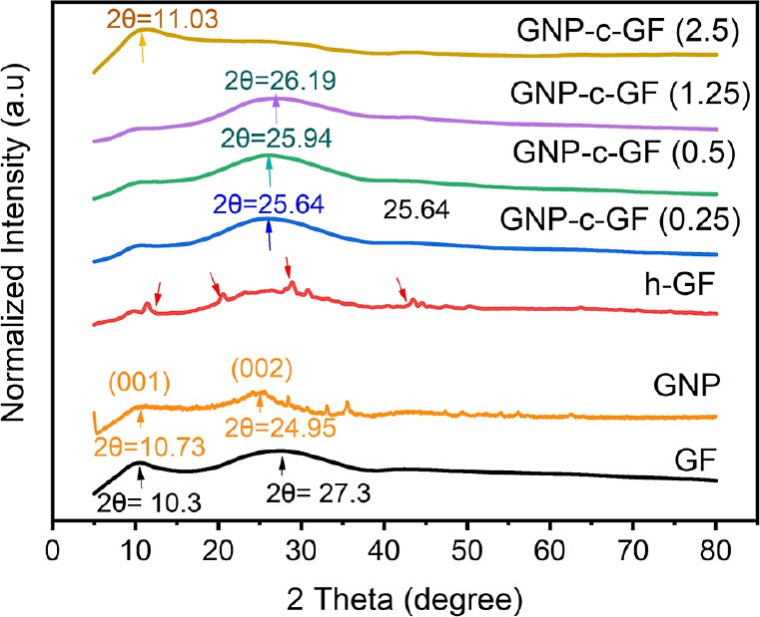
XRD patterns of the GF, GNP, h-GF, and GNP-c-GF materials.

XRD can be used to analyze GNP-c-GFs to gain insights into the structural properties of the material. Figure 9 displays the XRD patterns of the GF, h-GF, GNP, and GNP-c-GF materials. GNP with the (001) and (002) planes can be observed at 2θ = 10.3 and 2θ = 27.3, respectively. The GF sample exhibited a high and broad peak at 2θ = 27.3, indicating its amorphous structure.^31^ After hydroxylation (h-GF), the appearance of new peaks, indicated by red arrows, can be attributed to the etched surface due to the removal of surface organic groups and the attachment of new molecules. After coating with GNP, those peaks disappeared due to the existence of GNP on the surface for GNP-c-GF samples. The peak at 2θ = 27.3 slightly shifted to lower degrees for GNP-c-GF materials and the 002 peak of GNP^32^ merged with the peak at 2θ = 27.3 in GF, resulting in broader and more intense peaks. This observation provides evidence of the successful coating of GNP onto the h-GF surface. On the other hand, the same peak at around 2θ = 27.3 in GNP-c-GF (2.5) exhibited a distinctive behavior compared to other coatings, with a decrease in both peak width and intensity while the peak at 2θ = 11.03 became more noticeable. This pattern of GNP-c-GF (2.5) can be attributed to excessive GNP agglomeration, which creates surface heterogeneity on the fiber. Agglomeration of GNP can influence the surface roughness and continuity of the fiber, and this, in turn, may affect the XRD pattern. The discontinuity of the GNP coating resulting from agglomeration may lead to uncovered regions on the fiber surface, introducing variations in crystalline structures or the absence of graphene coating, ultimately contributing to the loss of specific peaks in the XRD pattern.

### 3.2 Effect of GNP-c-GF on the Mechanical Performance of PP Composites

**Figure 10:**
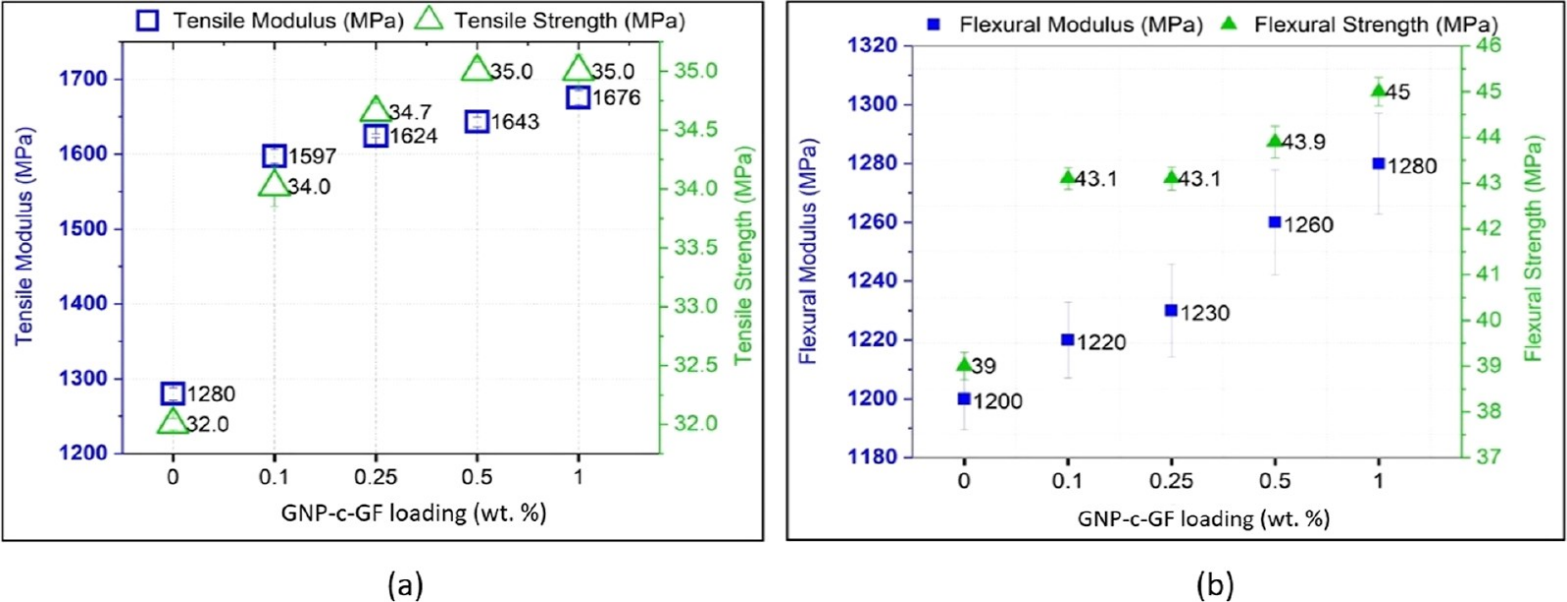
(a) Tensile and (b) flexural properties of PP/GNP-c-GF composites.

The evaluation of flexural and tensile properties plays a critical role in assessing the mechanical behavior and performance of composites. These properties offer valuable insights into how the material responds to bending and tensile forces, which are commonly encountered in real-world applications. By measuring these properties, we can determine the composite’s capacity to withstand applied forces, resist deformation, and maintain its structural integrity. Such measurements enable us to compare and select the appropriate composite materials that meet the performance requirements of specific engineering applications. The influence of GNP-c-GF on the mechanical properties of PP was investigated through tensile and flexural testing and various weight percentages (0.1–1%) of GNP-c-GF were incorporated into the PP matrix and shown in Figure 10. The findings demonstrated that even a minimal addition of GNP-c-GF at 0.1% by weight resulted in a notable enhancement of the tensile modulus. Specifically, there was a substantial 31% increase in the tensile modulus compared to neat PP when wt 1% of GNP-c-GF was introduced. A higher tensile modulus implies that the composite material is less prone to elongation and deformation under the applied tensile loads. This increased stiffness contributes to improved structural integrity, reducing the risk of deformation or failure under a load. While the flexural modulus values experienced a lesser degree of impact compared to the tensile modulus values, the inclusion of 1 wt % % GNP-c-GF exhibited a significant enhancement in flexural strength. This resulted in a remarkable 15% increase, reaching a value of 45 MPa. The primary disparity in the results between the tensile modulus and flexural modulus can be attributed to the elongated shape of the fibers and their heightened resistance when they are oriented in the tensile direction.

In the Supporting Information, Tables S1 and S2 provide a comparison of the mechanical properties of PP/GNP and PP/GNP-c-GF composites, which were examined as part of the benchmarking study. The composites containing GNP-c-GF exhibited higher values for tensile modulus, while PP/GNP composites surpass in flexural modulus across all concentrations. While the yield strength and flexural strength values were similar between the two types of composites, the ones containing GNP-c-GF showed slightly higher values. This indicates that the GNP coating on GF can enhance the mechanical properties of the composites even at lower concentrations, outperforming the use of GNP alone.

**Figure 11:**
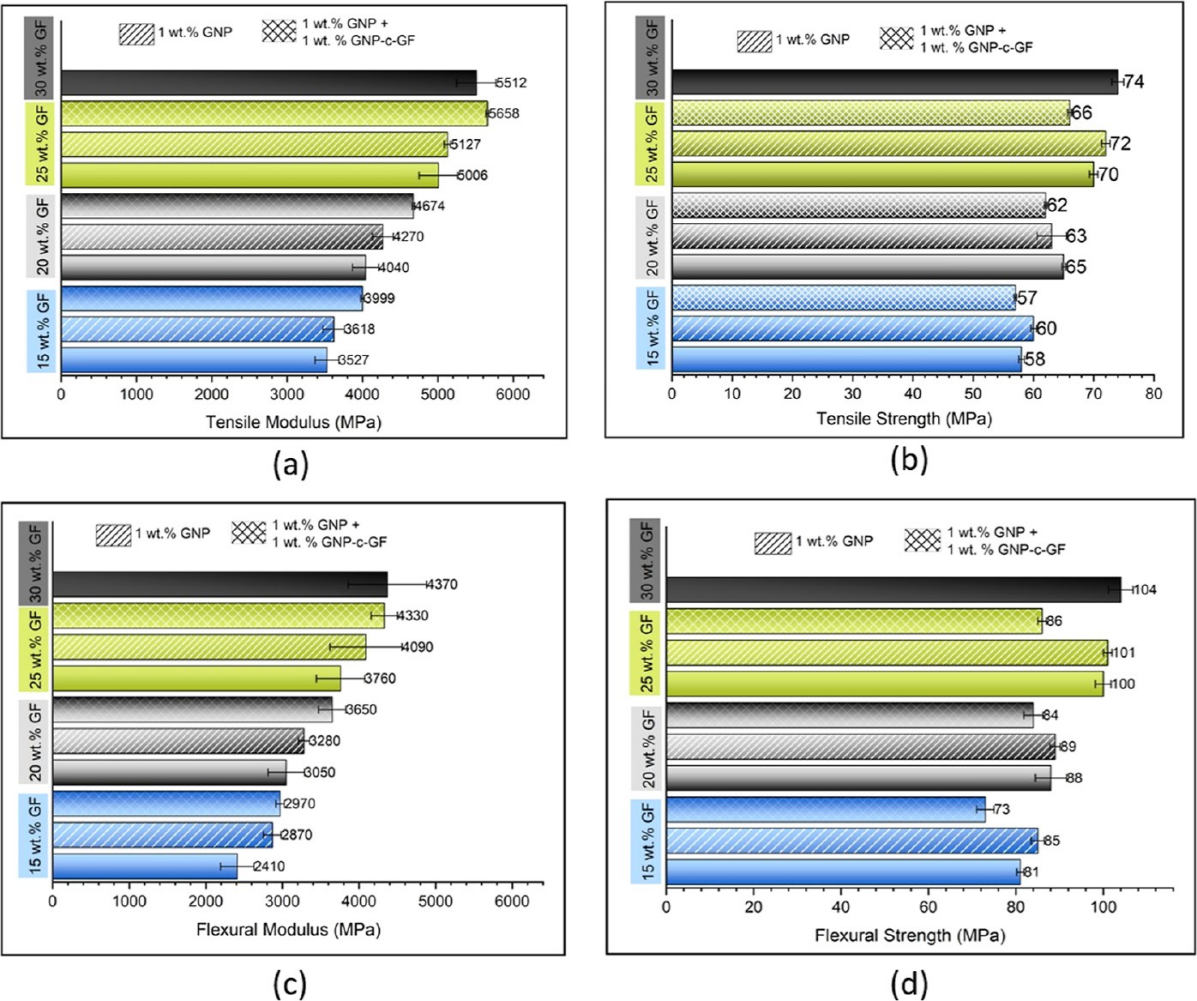
(a) Tensile modulus, (b) tensile strength, (c) flexural modulus, and (d) flexural strength of composites filled with 15% (blue), 20% (gray), 25%, and 30% GF, incorporating only GNP (striped) or a combination of GNP and GNP-c-GF (checkered).

Tensile properties are particularly important for applications where the material needs to withstand pulling or stretching forces, such as in structural components or load-bearing applications, and therefore GF reinforced composites are a perfect match for this kind of applications. The interaction between the fibers and the matrix material is critical for achieving high tensile properties. Figure 11 presents a comparative evaluation of the tensile and flexural properties of composites with varying compositions. The composites with different GF contents, namely, 15, 20, 25, and 30%, are denoted by the colors blue, gray, green, and black, respectively. All composites include GF, indicated on the *y*-axis. Solid columns represent GF-only composites, striped ones include both GF and GNPs, and checkered ones include GNPs and GNP-c-GF. Initially focusing on the tensile modulus of the GF/PP composites, the GF content was incrementally increased from 15 to 30% in the PP matrix, resulting in the expected enhancements starting from 3527 to 5512 MPa which can be attributed to the high aspect ratio and stiffness of the GFs. Furthermore, the introduction of 1 wt % GNP to the PP/GF composites led to additional improvements in the mechanical properties compared to the composites with the same GF content. Subsequently, with the incorporation of GNP-c-GF, a further increase in the tensile modulus was observed, surpassing the values achieved by the GF-only composites. The tensile modulus of the composite containing 30% GF was determined to be 5512 MPa, while the composite with 25% GF, supplemented with GNP and GNP-c-GF, exhibited a higher value of 5658 MPa. By using GNP-c-GF, the increase in surface roughness promotes a larger contact area between the fiber and matrix, leading to enhanced interfacial shear bonding through improved mechanical interlocking between the two components.^33^ This finding suggests the potential for attaining stronger composites with reduced GF content, highlighting the importance of a precise composite formulation. Moreover, a 5% reduction in the GF loading can offer significant advantages in terms of weight and density for the composite. Additionally, the incorporation of GNP-c-GF resulted in a considerable reduction in the standard deviation values associated with the mechanical properties of the composites. The assessment of the standard deviation is crucial in evaluating the consistency and homogeneity of results, indicating a more uniform distribution of properties throughout the composite. Calculated coefficient of variation values for composite samples are provided in Table S3. Notably, the GF-only composites exhibited elevated standard deviation values, whereas the addition of GNP-c-GF led to nearly identical tensile modulus results in five consecutive measurements. This observation underscores the achievement of enhanced filler dispersion and improved homogeneity facilitated by the incorporation of GNP-c-GF. Tensile strength is a fundamental mechanical property used to assess the maximum stress capacity of a material under tension, providing crucial insights into its structural integrity and reliability. A strong and well-bonded fiber–matrix interface ensures efficient stress transfer and prevents fiber pullout or debonding, leading to improved tensile strength. The analysis of tensile strength values depicted in Figure 11b reveals that the composite filled with 30% GF displayed the highest value of 74 MPa, aligning with expectations due to its elevated GF content. However, upon closer examination of composites with identical GF content, with the addition of GNP or GNP-c-GF, no discernible pattern of notable increase, decrease, or consistent trend is observed, suggesting that the tensile strength values do not exhibit significant changes. Notably, the standard deviation values of composites containing GNP-c-GF are much lower than those of the other composites. This reduction in standard deviation suggests a higher degree of homogeneity in both the obtained results and the overall behavior of the composites. Flexural modulus and flexural strength are important mechanical properties that provide insights into the structural behavior and performance of composite materials under bending loads. The flexural modulus represents the material’s resistance to deformation when subjected to a bending force, indicating its stiffness and ability to maintain its shape. The flexural modulus and flexural strength values of the produced composites can be seen in Figure 11c,d. Overall, when looking at composites with the same GF content, the addition of GNP and GNP-c-GF resulted in a gradual increase in the flexural modulus values. In particular, the GNP and GNP-c-GF-containing composite with 25% GF loading exhibits a value of 4370 MPa with a low standard deviation, which is very close to the 4370 MPa shown by the 30% GF-filled composite. When examining the tensile strength values, increasing strength values were observed with increasing GF content, and slight increases were obtained with the addition of GNP. This may be attributed to the resistive response of the GFs at certain points, as shown in SEM images in Figure 13a,c, which will be discussed in a related section later. When GFs cluster together, they can collectively generate a resistant force against applied stress at specific locations. After adding GNP-c-GF, slight decreases were observed, which suggests that the agglomerated fibers are separated by GNP-c-GF, leading to more homogeneous dispersion within the matrix and the disruption of the cohesive force between fibers.

**Figure 12:**
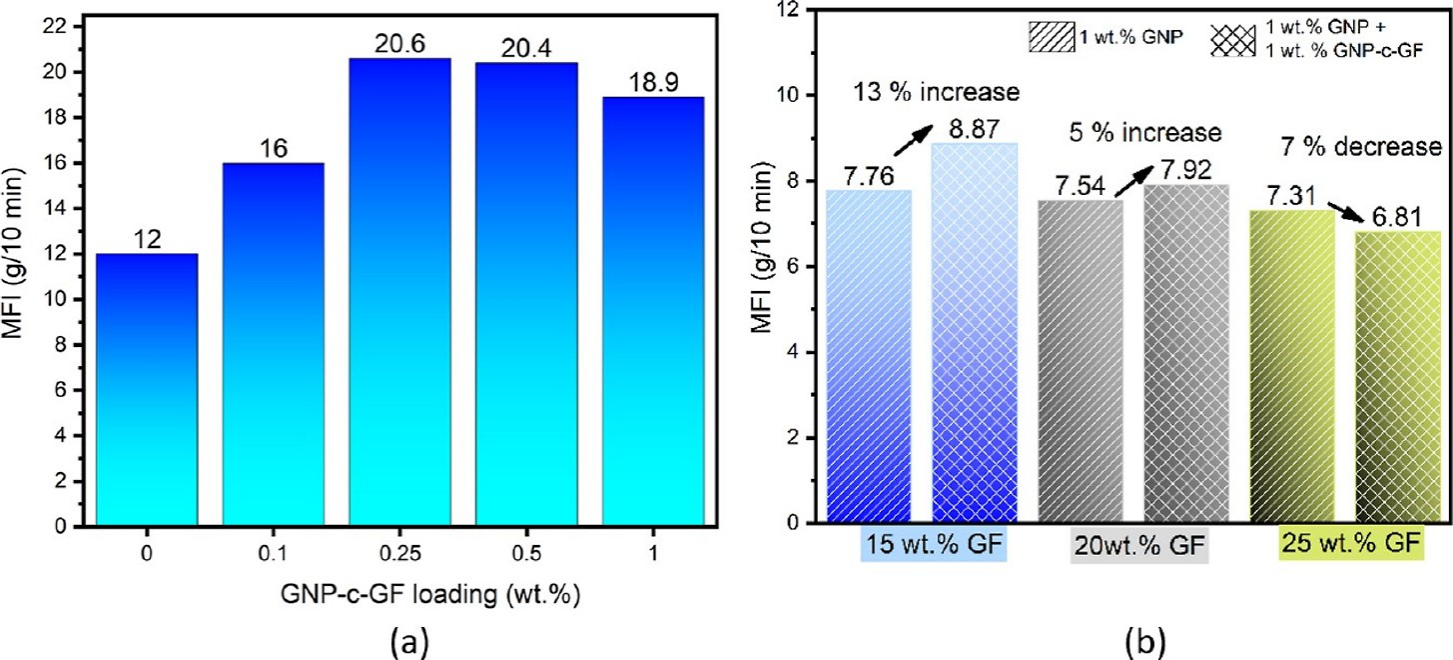
MFI values of (a) PP/GNP-c-GF composites and (b) composites filled with 15% (blue), 20% (gray), and 25% GF, incorporating only GNP (striped) or a combination of GNP and GNP-c-GF (checkered).

Melt flow index (MFI) serves as a crucial parameter, offering valuable insights into the viscosity and particle distribution characteristics of the materials. MFI values for two types of composites, namely, PP/GNP-c-GF and PP/GF/GNP/GNP-c-GF, are depicted in Figure 12a,b, respectively. Examination of the PP/GNP-c-GF composites revealed an increment in MFI values up to a loading ratio of 0.5 wt %, indicating an enhancement in flow properties. This improvement can be attributed to the oriented alignment of GFs in the flow direction and the concurrent presence of GNPs, which acted as a lubricant within the matrix. However, as the loading ratio reached 1%, a reduction in viscosity was observed due to the formation of an interconnected network structure among the incorporated GNP-c-GF particles. This network structure limited the mobility of the matrix, thereby effectively demonstrating the significant contribution of GNP-c-GF. The interfacial compatibility between the components was investigated by introducing GNP-c-GF into PP/GF/GNP composites. Specifically, the addition of GNP-c-GF to a GNP-reinforced composite with 15% GF loading resulted in a notable 13% increase in MFI, highlighting the improved interfacial properties. For the composite with 20% GF loading, the observed increase in MFI was reduced to 5%. These findings indicate that the inclusion of GNP-c-GF mitigated the restrictions imposed by rigid GFs, thereby promoting better interfacial compatibility and exhibiting a lubricating effect within the matrix. Moreover, in the composite with 25% GF loading, the added GNP-c-GF achieved a sufficient level of saturation and facilitated the formation of a network structure among the particles. Consequently, this network structure impeded the flow of the matrix, leading to a 7% reduction in MFI. The decrease in MFI value resulted in an increase in viscosity, reflecting the altered rheological behavior induced by the incorporation of GNP-c-GF. Hence, the experimental results highlight the favorable influence of GNP-c-GF on the melt flow properties, interfacial compatibility, and viscosity of the composites, shedding light on their potential for advanced applications in various fields.

### 3.3 Effect of GNP-c-GF on the Morphological Analysis of PP/GF/GNP Composites by SEM

**Figure 13:**
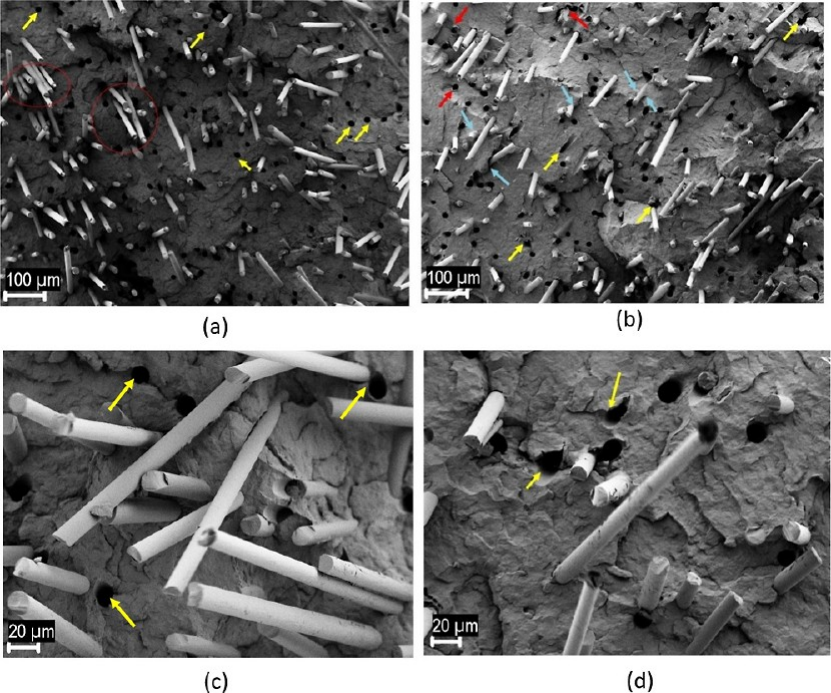
SEM images of (a,c) PP/GF/GNP composites and (b,d) PP/GF/GNP/GNP-c-GF composites at different magnifications.

SEM is a highly valuable tool in composite research as it enables the examination of the composite’s microstructure with exceptional detail and resolution. By analysis of the morphology, distribution, and alignment of the reinforcing fibers or particles in the composite matrix, SEM provides essential insights into the interfacial interactions, bonding, and overall performance of the composite. This information is crucial for understanding how the composite behaves under various conditions and aids in optimizing its design and manufacturing processes. Figure 13 presents morphological analysis of the effect of GNP-c-GF on the 20% GF and 1% GNP filled composites. Composites filled with 20% GF have black cavities, indicating fiber break points, giving some clues as to the way the fiber breaks from the matrix. For example, the rounds from which the fibers in Figure 13a come out are almost perfectly round (yellow arrows), indicating that the GFs break without difficulty as they pull out of the matrix. On the other hand, the round shape of most of the circles in Figure 13b has been distorted, and this homogeneity has been disrupted by taking an elliptical structure or forming an angular shape at some points, as indicated by the yellow arrows. This proves that at some points, it is more difficult for the fibers to separate from the matrix. With the red arrows, deformed matrix can be seen on the edges of the circles. Contrary to Figure 13a, at the points indicated by the blue arrows in b, the bond of the fibers with the matrix is clearly visible due to the polymer matrix adhered to the fiber. Therefore, it can be said that matrix and fiber adhesion become stronger with GNP-c-GF content. In the composite without GNP-c-GF, it is also seen that a few fibers agglomerate at the points indicated by the red circle. Closer images also appear in Figure 13c,d. Here, again, there is a gap of three fibers that have been agglomerated from the matrix, which is indicated by a red circle. The smooth circles (yellow arrows) of the broken fibers also appear more clearly. In Figure 13d, the deformation of the cavities where the fibers have come out is also shown more closely with yellow arrows. Overall, it is possible to say that increased fiber–matrix compatibility is achieved at some points in the presence of GNP-c-GF in the environment.

## 4 Conclusions

The current study employed a facile and practical coating technique to treat GFs with upcycled GNPs derived from waste tires. Incorporating unmodified GNPs and conducting an optimization study on dip coating resulted in the successful production of GNP-coated GFs (GNP-c-GF). These GNP-c-GF were effectively integrated into pure PP and PP/GF/GNP composites, leading to substantial improvements in their mechanical, morphological, and flow properties. Notably, the inclusion of 1 wt % GNP-c-GF in neat PP resulted in a significant 31% increase in tensile modulus compared to pure PP. Moreover, 25 wt % GF filled PP, with the addition of GNP-c-GF and GNP, exhibited a higher value of 5658 MPa, while 30% GF filled PP had a tensile modulus of 5512 MPa. Furthermore, the addition of GNPs and GNP-c-GF resulted in increased flexural modulus values in composites with the same GF content. The tensile strength values did not exhibit significant changes with the addition of GNPs or GNP-c-GF. However, the standard deviation values of composites containing GNP-c-GF were significantly lower, indicating improved homogeneity. MFI analysis showed that the incorporation of GNP-c-GF enhanced the flow properties of the composites up to a loading ratio of 0.5 wt %. However, at higher loading ratios, a network structure formed among the GNP-c-GF particles, reducing viscosity and limiting the mobility of the matrix. The presence of GNP-c-GF also improved the adhesion between fibers and the matrix, as observed in the morphology analysis. Reduced fiber agglomeration led to a more homogeneous dispersion within the matrix, disrupting the cohesive forces between fibers.

### 4.1 Future Perspectives

The current study opens up several avenues for future research and development in the field of GNP-c-GF composites. While the current study utilized a practical dip coating technique for GNP-c-GF production, further optimization and refinement of the coating process can be explored. Fine-tuning parameters, such as coating time, temperature, and concentration, could potentially enhance the coating efficiency and ensure uniform dispersion of GNPs on the GF surface. Future investigations can delve into other mechanical properties, such as impact strength, fatigue resistance, and creep behavior. Exploring the effects of different GNP-c-GF loadings and fabrication techniques on these properties would contribute to a more comprehensive understanding of the composite’s performance. The influence of processing parameters on the dispersion and alignment of GNP-c-GF within the matrix can be explored further. Investigating alternative processing techniques, such as injection molding or extrusion, and their impact on the composite properties would aid in optimizing the manufacturing process. The successful integration of GNP-c-GF in this study provides valuable insights and suggests several potential future applications and benefits. GNP-c-GF composites can be explored as lightweight alternatives in various industries such as automotive, aerospace, and construction. Their improved mechanical properties and compatibility could enable the design of lightweight components with enhanced strength and stiffness. The utilization of upcycled GNPs derived from waste tires in this study aligns with the growing demand for sustainable materials. Further research could explore the environmental impact and life cycle analysis of GNP-c-GF composites, highlighting their potential as ecofriendly alternatives in various industries. Further exploration of the scalability and feasibility of the GNP-c-GF production process is crucial. Investigating the potential for large-scale manufacturing and cost-effectiveness would facilitate practical implementation of these composites in industrial settings.

## Supporting Information Available

Experimental stages of GF coating process, Raman spectra of coating materials, comparative mechanical results of PP/GNP and GNP-c-GF/PP composites, and calculated coefficient of variation values for composites (PDF)
