# Supplementary material for: Utilizing Upcycled Graphene Nanoplatelet-Coated Glass Fibers as a Performance Booster and Compatibilizer for Enhanced Mechanical Performance of Polypropylene/Glass Fiber/Graphene Nanoplatelet Composites
Source: ACS Omega. 2024 Apr 2;9(15):17432–45. doi: 10.1021/acsomega.4c00214 (PMC11024938; doi:10.1021/acsomega.4c00214)
Supplement: Supplementary file 1 — ao4c00214_si_001.pdf [file ao4c00214_si_001.pdf]

SUPPORTING INFORMATION

Utilizing Upcycled Graphene Nanoplatelet-Coated Glass Fibers as a Performance Booster and Compatibilizer for Enhanced Mechanical Performance of Polypropylene/Glass Fiber/Graphene Nanoplatelet Composites

Gülayşe Şahin Dündar [1,2], Burcu Saner Okan [1,2,*]

[1] Department of Materials Science and NanoEngineering, Faculty of Engineering and Natural Sciences, Sabanci University, Orhanli-Tuzla, 34956, Istanbul, Turkey

[2]Sabanci University Integrated Manufacturing Technologies Research and Application Center & Composite Technologies Center of Excellence, Teknopark Istanbul, 34906, Pendik, Istanbul, Turkey

*Corresponding author: burcu.saner@sabanciuniv.edu

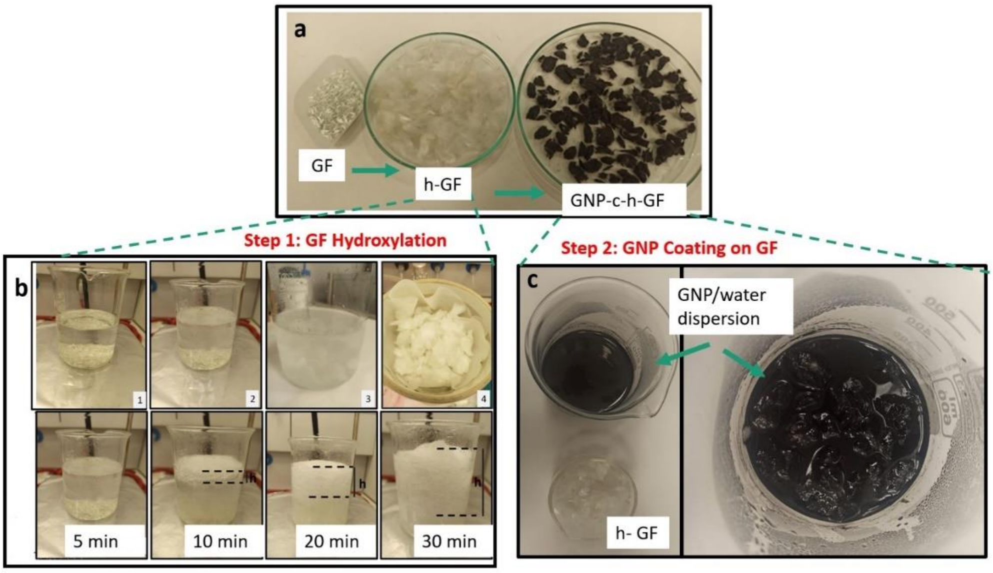

Figure S1. Experimental stages of GF coating process showing (a) materials, (b) desizing and hydroxylation process of GF, and (c) dip coating of GF in GNP/water dispersion

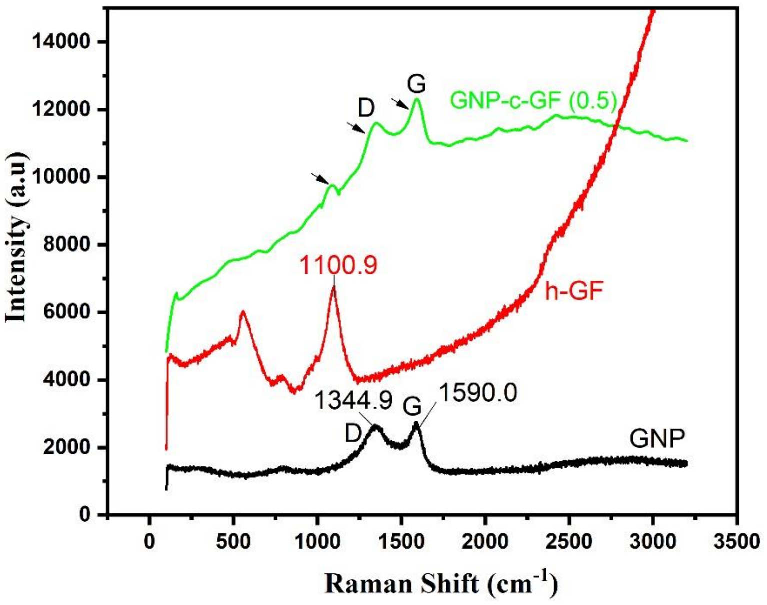

Figure S2. Raman spectra were recorded for the GNP (black), h-GF(red), and GNP-c-GF (0.5) (green). Asymmetric stretching mode of Si-O-Si at 1100.9 cm^{-1} , and D and G peak of GNP is found on the GNP-c-GF (0.5) sample indicating the successful coating of GNP on the fiber surface.

Table S1. Comparative mechanical results of PP/GNP and GNP-c-GF/PP composites at a loading of 0.1 wt% -1 wt% by twin screw extruder.

Sample Name	0.1 wt% GNP	0.1 wt% GNP-c-GF	0.25 wt% GNP	0.25 wt% GNP-c-GF	0.5 wt% GNP	0.5 wt% GNP-c-GF	1 wt% GNP	1 wt% GNP-c-GF
Tensile Modulus (MPa)	1440	1597	1444	1624	1512	1643	1485	1675
Tensile Strength (MPa)	33	32	33	34	33	35	33	35
Flexural Modulus (MPa)	1250	1220	1300	1230	1310	1260	1330	1280
Flexural Strength (MPa)	42	43	43	43	44	44	44	45

Table S2. Mechanical improvement (%) of PP/GNP and GNP-c-GF/PP composites compared to neat PP at a loading of 0.1 wt% -1 wt% by twin screw extruder.

Sample Name	0.1 wt% GNP	0.1 wt% GNP-c-GF	0.25 wt% GNP	0.25 wt% GNP-c-GF	0.5 wt% GNP	0.5 wt% GNP-c-GF	1 wt% GNP	1 wt% GNP-c-GF
Tensile Modulus Improvement (%)	13	25	13	27	18	28	16	31
Tensile Strength Improvement (%)	3	0	3	6	3	9	3	9
Flexural Modulus Improvement (%)	4	2	8	3	9	5	11	7
Flexural Strength Improvement (%)	8	10	10	10	13	13	13	15

Table S3. Calculated coefficient of variation (CV) values for composite samples

Sample Name	CV for tensile modulus	CV for tensile strength	CV for flexural modulus	CV for flexural strength
PP/ 15%GF	0.044514	0.008103	0.090456	0.009877
PP/ 15%GF/ GNP1%	0.039248	0.007333	0.040767	0.017647
PP/ 15%GF/ GNP1%/GNP-c-GF 1%	0.005901	0.003509	0.018519	0.027397
PP/ 20%GF	0.043069	0.004923	0.077705	0.040909
PP/ 20%GF/ GNP1%	0.032553	0.038095	0.022256	0.013483
PP/ 20%GF/ GNP1%/GNP-c-GF 1%	0.004065	0.003226	0.049041	0.02619
PP/ 25%GF	0.050739	0.01	0.083777	0.018
PP/ 25%GF/ GNP1%	0.007997	0.009722	0.115403	0.009901
PP/ 25%GF/ GNP1%/GNP-c-GF 1%	0.003181	0.004545	0.039492	0.011628
PP/ 30%GF	0.047533	0.013514	0.117391	0.026923
